# Spatial resolution of confocal XRF technique using capillary optics

**DOI:** 10.1186/1556-276X-8-271

**Published:** 2013-06-07

**Authors:** Maël Dehlinger, Carole Fauquet, Sebastien Lavandier, Orawan Aumporn, Franck Jandard, Vladimir Arkadiev, Aniouar Bjeoumikhov, Didier Tonneau

**Affiliations:** 1 CNRS, UMR7325, Aix-Marseille Univ., CINaM, Marseille 13288, France; 2 IFG-GmbH, Rudower Chaussee 29/31, Berlin 12489, Germany

**Keywords:** X-ray fluorescence, Polycapillary, Monocapillary

## Abstract

XRF (X-ray fluorescence) is a powerful technique for elemental analysis with a high sensitivity. The resolution is presently limited by the size of the primary excitation X-ray beam. A test-bed for confocal-type XRF has been developed to estimate the ultimate lateral resolution which could be reached in chemical mapping using this technique. A polycapillary lens is used to tightly focus the primary X-ray beam of a low power rhodium X-ray source, while the fluorescence signal is collected by a SDD detector through a cylindrical monocapillary. This system was used to characterize the geometry of the fluorescent zone. Capillary radii ranging from 50 μm down to 5 μm were used to investigate the fluorescence signal maximum level This study allows to estimate the ultimate resolution which could be reached in-lab or on a synchrotron beamline. A new tool combining local XRF and scanning probe microscopy is finally proposed.

## Background

X-ray fluorescence (XRF) is a highly sensitive, non-destructive technique that is able to detect element traces for material elemental analysis. It is now widely used in various fields of science such as material processing [1], cultural patrimony [2], archaeology [3], medical and biology [4], environment [5], etc. Two approaches are possible to increase the XRF lateral resolution for chemical mapping. First, the primary probe diameter can be decreased as the detector aperture is increased to keep a significant signal-to-noise ratio. This is the general tendency both for in-lab classical XRF and in synchrotron environment where 30-nm resolution can be offered on few beamlines (see example in [6]). The second solution consists in keeping the primary beam diameter constant and decreasing the detector input aperture. In this latter case, it must be approached as much as possible towards the surface to keep a significant XRF signal detection. However, the detector steric hindrance impedes approaching at sub-millimetre distance from the surface without primary beam shadowing. A solution is to use a sharp monocapillary to collect the XRF signal near the surface. The XRF signal is proportional to the primary source brightness and thus, in both modes, the higher is the brightness, the higher the signal-to-noise ratio can be expected.

Thanks to the development of new focusing optics like polycapillary lens [7,8], micro-XRF analysis became possible using laboratory and even portable X-ray sources [9]. In this case, the lateral resolution of the technique is essentially provided by the primary beam geometry and still leads to numerous works in a huge variety of domains [1,10]. Later, equipping the detector with a second polycapillary lens, a new concept based on a confocal configuration was proposed. Indeed, the detected signal comes from the intersect between the volume excited nearby the source lens focal plane and the analyzed volume in the vicinity of the detector lens focal plane [11-15]. The spatial resolution of the confocal micro-XRF technique is thus enhanced compared to the classical configuration.

However, it is possible to further enhance the spatial resolution of the technique, further shrinking the detector acceptance, and approaching virtually towards the surface using a thin cylindrical capillary. In this work, we have built a test-bed for feasibility demonstration using single cylindrical glass capillaries of 50- down to 5-μm radius equipping an EDX detector. XRF escaping from a Co sample irradiated by a focused micro-X-ray source was measured by these means. From the detected flux values, extrapolation gave low flux values that should be realistically measurable with the same detector equipped with a 0.5-μm radius cylindrical capillary.

## Methods

**Figure 1:**
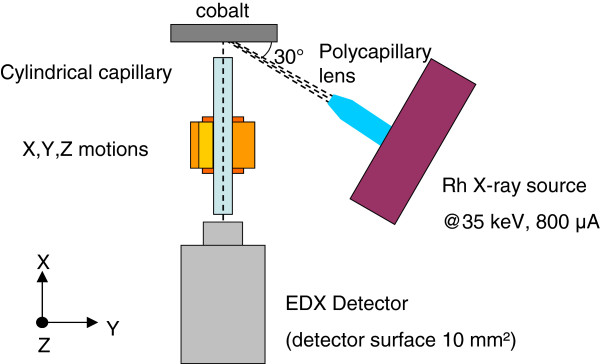
Principle of the confocal XRF test bed used in this study.

The experimental setup of the confocal XRF test-bed is shown in Figure 1. An X-ray beam provided by a low power Rh source operating at 35 kV and 800 μA is focused on a sample using a 6-mm focal distance polycapillary lens [16,17]. The beam incidence angle is 30°. The source spectrum exhibits a wide Bremsstrahlung radiation, narrow Rh-K_α_, Rh-K_β1_ and Rh-K_β2_ rays at 20.216, 22.074 and 22.724 keV, respectively, and X-rays from the L shell excitation at 2.697, 2.692, 2.834, 3.001 and 3.144 keV. Bremsstrahlung, K_α_, K_β_ and sum of X-ray radiation from the L-edge is respectively 56.23%, 2.67%, 0.62% and 40.48% of the total photon flux at 35 kV electron acceleration voltage on (using) a rhodium target [18]. The sample fluorescence is collected by SDD (silicon drift detector, Brüker GmbH, Karlsruhe, Germany; surface 10mm^2^) and EDX (energy dispersive X-ray) detector through a 50-mm long and 1-mm outer diameter cylindrical X-ray monocapillary. The capillary inner radius is 5, 10, 25 or 50 μm. The cylindrical capillary is placed on X, Y, Z piezo-stages allowing displacements with 30-nm step size while the detector remains in a fixed position. The capillary extremity to sample distance (i.e. the working distance, WD) is fixed at 1 mm for all experiments. The signal collected depends on the solid angle under which the capillary aperture is seen from the fluorescence zone. Thus, this parameter has to be kept constant during capillary replacement procedure. The 1-mm value is controlled by placing the capillary in contact with the surface and by removing it using the Z-motion. One millimetre is a high enough WD to avoid primary beam shadowing effect by the capillary nozzle. A cobalt sample, exhibiting a significant X-ray fluorescence yield under the Rh source excitation, is used to measure the fluorescence signal magnitude collected through the different cylindrical capillaries and to estimate the fluorescence flux which could be collected with thinner capillaries.

## Results and discussion

**Figure 2:**
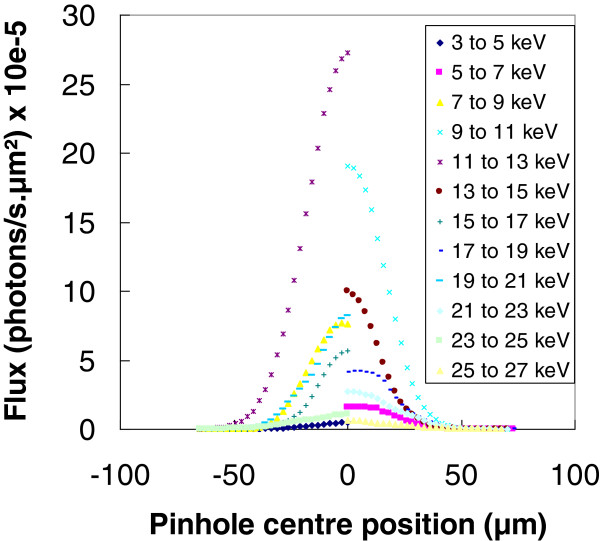
Lateral photon flux profile for different X-ray energy ranges.

In the first series of experiments, the primary spot was characterized. For that purpose, the detector is positioned in direct view of the primary beam. The detector entry is shrunk using a 5-μm diameter lead pinhole placed on the X, Y, Z piezo stages. The pinhole is positioned in the polycapillary lens focal plane and is displaced along the beam spot diameter in the same plane. For each pinhole position, a primary beam spectrum is acquired. Figure 2 shows the X-ray photon flux variations with the pinhole centre position within different incident energy ranges. The incident spot profile has a Gaussian shape, and the radius as well as the maximum flux depends on the photon energy. The lens providing the spot consists in a monolithic system made of a great number of monocapillary micrometric glass tubes bent together [10]. Because the Rh low power source is not monochromatized, the total external reflection critical angle of glass *θ*_c_ should vary with source energy *E* in agreement with the following equation:

(1) $$\theta_{c}=\frac{0.02\;\sqrt{\rho }}{E},$$

where *ρ* is the glass capillary density. This is the reason why the incident spot radius provided by the polycapillary lens depends on the photon energy range, as can be seen in Figure 2. The average spot radius measured at 1/e is 22 μm, and the total photon flux within this spot area is about 1.7 × 10^9^ photons/s.

**Figure 3:**
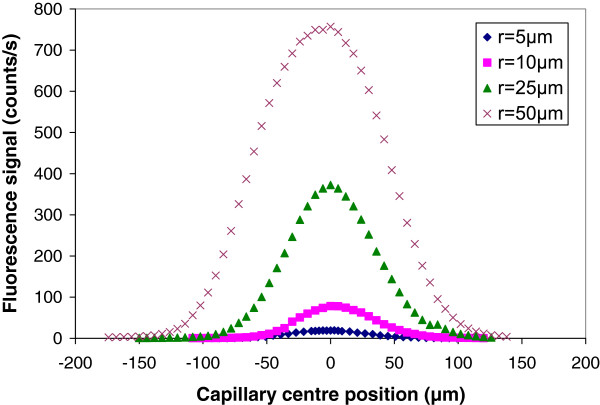
**Fluorescence zone profile.** The cobalt sample is placed in the focal plane of the polycapillary lens used to focus the rhodium source beam. The capillary inner radius is 5, 10, 25 or 50 μm.

**Figure 4:**
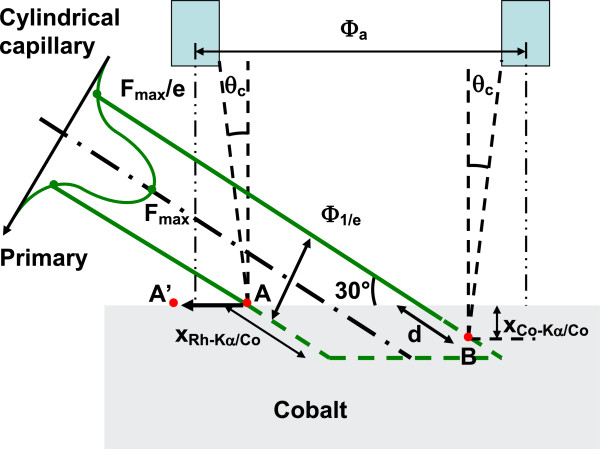
Sample excited volume geometry.

Then, the geometry of the fluorescence emitting volume in the cobalt sample was defined using the confocal XRF configuration shown in Figure 1 by scanning the cylindrical capillary used for detection along the X-ray fluorescence emitting zone. At each cylindrical capillary position, an X-ray spectrum is acquired that exhibits the two characteristic Co-K_α_ and Co-K_β_ lines at 6.9 and 7.6 keV, respectively. We then reported in Figure 3 the K_α_ peak area measured for each capillary position using various capillary radii from 5 to 50 μm. All the curves exhibit identical shape which are not expected to be Gaussian. The primary beam is not perpendicular to the surface so that it penetrates inside the sample with an attenuation length x_Rh-Kα/Co_ = 43 μm [19] inducing X-ray fluorescence, itself reabsorbed and leading to secondary emission. This means that the collected fluorescence comes from a deep excited volume schematically shown in Figure 4. However, the fluorescence emitted within this deep volume cannot be entirely detected since the attenuation length of Co-K_α_ rays in Co (x_Co-Kα/Co_ = 18 μm [19]) is shorter than the penetration depth of Rh-K_α_ rays in Co. From simple geometrical considerations, neglecting the secondary emission, a signal is detected over a capillary travel *Φ*_a_ given:

(2) $$\begin{array}{l} \Phi_{a}=2\mathrm{WD}\tan\left(\theta_{c}\right)+{2r}_{\mathrm{spot}}/\sin\left(30\right)\\ \;+x_{\mathrm{Co}-K\alpha /\mathrm{Co}}\cot\left(30\right)+{2r}_{\mathrm{cap}} \end{array}$$

where *r*_spot_ is the primary spot radius measured at 1/e, *r*_cap_ is the capillary radius and WD is the detection capillary working distance. However, as can be seen in Figure 4, the fluorescence magnitude collected from point A, located at the cobalt sample surface, is obviously different from that collected from in-depth point B. This is due to the absorption of the primary beam before reaching point B and to strong fluorescence reabsorption in the path through the sample. Thus, in order to compare the theoretical and experimental values of *Φ*_a_, we must consider this discrepancy. Taking into account the actual value of the primary beam flux *F*_max_/e at *r*_spot_ from the spot centre (see Figure 4), the fluorescence maximum flux *F* (B) escaping from the sample emitted at a depth of x_Co-Kα/Co_ = 18 μm (point B)? should be given by:

(3) $$F\left(B\right)=F_{\max}\tau /e^{2}\;\exp\left(-d\;/\;x_{\mathrm{Rh}-K}{}_{\alpha /\mathrm{Co}}\right)$$

where *d* is the path length of the primary beam in Co till a depth of x_Co-Kα/Co_ and *τ* is the total fluorescence yield of Cobalt. With the value of *τ* = 33% taken from [19] the value of *F*(B) is expected to be about 0.02 *F*_max_. From this, we arbitrary choose the significant fluorescence flux above 0.02 *F*_max_ to define the capillary travel *Φ*_a_ along which fluorescence was detected from the sample surface. Point A’ must thus be chosen instead of point A, to fit with this condition:

(4) $$F\left(A’\right)=F_{\max}\exp-\left(r_{A’}{}^{2}\;/\;r_{\mathrm{spot}}{}^{2}\right)\tau =0.0{2\;F}_{\max}$$

Consequently, point A’ in Figure 4 is positioned at a distance *r*_A’_ = 1.7 *r*_spot_ from the beam centre. To compare the expected and measured values of *Φ*_a_, we have thus replaced 2 *r*_spot_ in Equation 1 by distance A’B = 1.7 *r*_spot_ + *r*_spot_. With these considerations, *Φ*_a_ values of 258, 208, 178 and 168 μm are expected for a capillary radius of 50, 25, 10 and 5 μm, respectively. These values are in good agreement with the experimental values of *Φ*_a_ = 240, 205, 172 and 168 μm.

**Figure 5:**
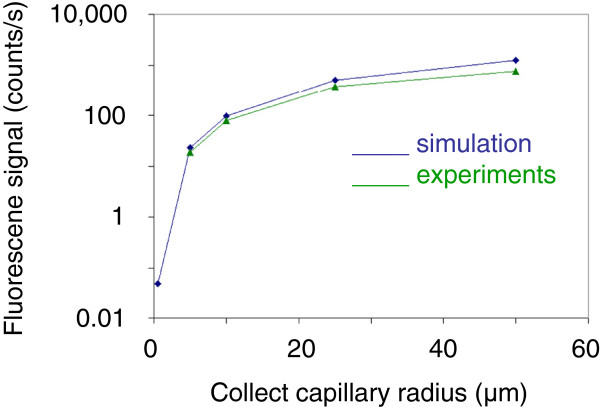
**Maximum fluorescence flux dependence on the capillary radius during capillary scan.** Experimental and simulated data.

**Figure 6:**
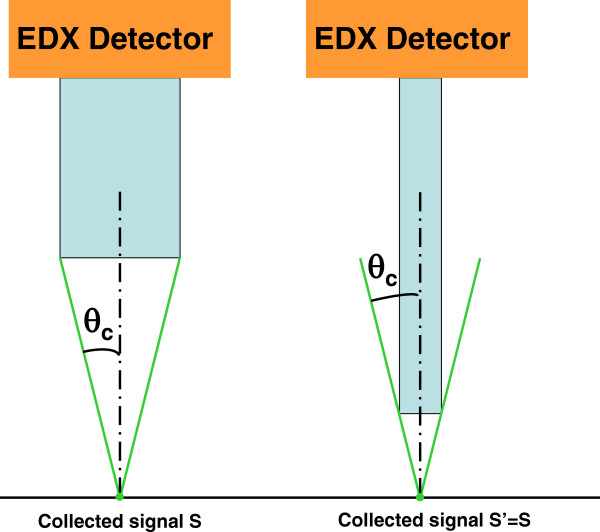
**X-ray collection using cylindrical monocapillary.** Dependence of the collected flux on capillary radius and length. In both configurations, the signal magnitude is the same.

We have then reported in Figure 5 the variations of the maximum flux collected at the centre of the fluorescent zone as a function of capillary radius for a constant WD of 1 mm. The maximum collected flux increases as r_cap_^1.8^. This variation has to be compared to the ideal case of fluorescence collection from a point source using a thin capillary of length L placed at a working distance WD from the emitter. Figure 6 clearly shows that the collected signal level should remain constant if the capillary radius is reduced, providing the WD is reduced by the same factor by increasing the capillary length and assuming an ideal transmission coefficient of 100%. Obviously, the capillary only collects a part of fluorescence, nearly proportional to its section. In our case, the observed variations of the signal magnitude with the capillary radius are due to the fact that the fluorescent zone has dimensions higher or of the same order of magnitude than the capillary radius.

Is it possible to increase this signal by decreasing WD?

**Figure 7:**
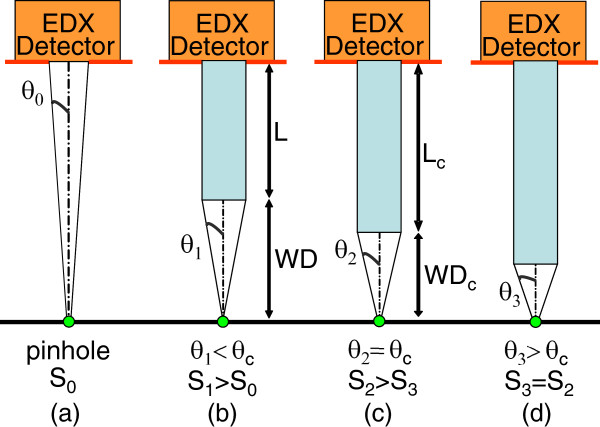
**X-ray collection using cylindrical monocapillary.** Dependence of the collected flux on capillary working distance WD at constant sample detector distance. The detection through a capillary increases the collection solid angle. (**a**) Detection through a pinhole. For short capillary length (**b**), the signal magnitude *S*_1_ is higher than *S*_0_ detected in case (**a**); (**c**) if WD is shortened until WD_c_, the signal magnitude *S*_2_ increases until *θ*_2_ = *θ*_c_; (**d**) for WD lower than WD_c_, the signal remains constant.

It is well known that cylindrical capillaries allow to significantly increase the collected signal by comparison with a pinhole with the same radius placed at the detector entry and positioned at the same WD + *L* distance (Figure 7a,b) [10]. At high WD, the capillary nozzle is seen under a solid angle *θ*_1_ <*θ*_c_ from a point source (Figure 7b). Thus, all X-rays emitted by the point source within this solid angle will be transmitted through the capillary, assuming a total reflection of X-rays below the critical angle. The capillary gain *G* regarding a pinhole of the same radius is given by the equation [10]:

(5) $$G\approx {\left[\theta_{1}\left(\mathrm{WD}+L\right)/r_{\mathrm{cap}}\right]}^{2}$$

If WD decreases, keeping WD + *L* constant, the collected signal magnitude first increases since the collection solid angle increases until it reaches *θ*_2_ = *θ*_c_ value. At this point (Figure 7c), WD reaches WD_c_ value given by:

(6) $${\mathrm{WD}}_{c}=r_{\mathrm{cap}}/\tan\left(\theta_{c}\right)\approx r_{\mathrm{cap}}/\theta_{c}$$

In this case, the capillary gain is given by:

(7) $$G={\left[\theta_{c}\left({\mathrm{WD}}_{c}+L_{c}\right)/r_{\mathrm{cap}}\right]}^{2}={\left[1+\theta_{c}L_{c}/r_{\mathrm{cap}}\right]}^{2}$$

If WD is further decreased, the solid angle *θ*_3_ under which the capillary nozzle is seen from the point source is higher than *θ*_c_ (Figure 7d). The collected signal is no more limited by the capillary acceptance: the capillary gain as well as the collected signal remain constant. Because the WD_c_ value depends on the capillary radius and the smallest value of WD_c_ is 1 mm for the capillaries tested in this work, this optimum value was chosen and taken constant in all these experiments.

Because the fluorescent emitting source in the experiments is not punctual, we have started simulations to estimate the flux collected with a 0.5-μm radius capillary positioned at a WD of 1 mm. These simulations are based on a finite element method calculation from fundamental parameter equations and will be presented elsewhere. Figure 5 shows the dependence of the collected signal with the capillary radius in the range of 0.5 to 50 μm. The calculated values are in good agreement with the experimental ones. The estimated flux with a 0.5-radius capillary is 0.07 photons/s. This value is obtained at 1 mm WD. However, the maximum signal should be reached at 100 μm WD_c_ value. For this WD_c_ value, about 0.7 counts/s flux can be expected. Note that increasing the acquisition time should lead to significant signal level enhancement with our EDX-SDD device. These results show that it is possible to collect the fluorescence signal using a thinner capillary without any loss on the signal level if it is close enough to the surface. Of course, using a brighter primary source such as a rotating anode or a liquid-metal jet anode electron-impact X-ray source [20], a significantly higher signal (up to 100 times) can be expected Moreover, replacing the cylindrical capillary at the entry of the detector by an elliptical one would lead to an extra gain of 20 [21,22]. Thus sub-micro-resolution XRF would be possible with an in-lab excitation source. Of course, working with a synchrotron source would lead to higher signal magnitude which could allow to further shrink the capillary radius, and a sub-100-nm lateral resolution could probably be reached. The short capillary-sample working distance suggests that the cylindrical capillary could act as a scanning probe microscope tip to acquire simultaneously sample topography and chemical mapping by XRF analysis [23], as already demonstrated for simultaneous SNOM-XAS XEOL [17] apparatus. Moreover, within this perspective, the spatial resolution of the detection would not be limited by the critical angle *θ*_c_ because the extremity of the glass tube would be approached in mechanical near-field interaction with the sample.

## Conclusions

In this work, we have developed a test-bed consisting in a low power Rh-source focused with a polycapillary lens on a cobalt sample and in a cylindrical capillary to collect the fluorescence signal at the vicinity of the surface. Both capillaries are positioned in a confocal-like configuration. The primary beam has been first characterized, and the lateral profile of the X-ray spot was found to be a Gaussian which radius and magnitude depend on the X-ray energy range. The average radius measured at 1/e is 22 μm. Then, a cobalt sample was placed in the focal plane of the lens, and the generated fluorescence was collected through a cylindrical capillary fixed on a SDD EDX dectector. The thin detection capillary was then scanned across the sample fluorescence emitting zone. Significant signal was collected over a total capillary travel in very good agreement with what can be deduced from simple geometrical considerations. The fluorescence signal magnitude increases as *r*_cap_^1.8^ where *r*_cap_ is the capillary radius. The extrapolated value for a 0.5-μm radius capillary suggests that sub-1-μm resolution XRF should be possible with a laboratory source. Of course, increasing the source brightness, i.e. working with liquid-metal or synchrotron sources could probably lead to reach 100-nm resolution. Operating at short working distances will allow the increase of the signal level detection. Furthermore, it could lead to a new generation of instrument, coupling XRF and scanning probe microscopy, allowing to simultaneously combine chemical analysis of a sample and topography.

## Competing interests

Patent concerning the detection of XRF through capillary optics is pending (European patent # PCT/IB2011/052423, 2011). The authors declare that they have no competing interests.

## Authors’ contributions

MD and OA carried out the experiments. SL and FJ were involved in instrument design, fabrication and calibration. MD, VA and DT carried out the simulations. CF, AB and DT participated in data interpretation and discussion. DT coordinated this study. MD, CF and DT drafted the manuscript. All authors read and approved the final manuscript.
